# Drivers of vaccination preferences to protect a low-value livestock resource: Willingness to pay for Newcastle disease vaccines by smallholder households

**DOI:** 10.1016/j.vaccine.2018.11.058

**Published:** 2019-01-03

**Authors:** Zoë A. Campbell, Linus Otieno, Gabriel M. Shirima, Thomas L. Marsh, Guy H. Palmer

**Affiliations:** a Paul G. Allen School for Global Animal Health, Washington State University, PO Box 647090, Pullman, WA 99164, USA; b Kenya Medical Research Institute, Kisumu, Kenya; c Nelson Mandela African Institution of Science and Technology, Arusha, Tanzania; d School of Economic Sciences, Washington State University, Pullman, WA, USA

**Keywords:** Vaccination decision, Contingent valuation, Willingness to pay, Veterinary vaccines, Poultry, Food security

## Abstract

Vaccination can be an effective risk management approach to minimize the burden of disease and increase livestock productivity for smallholder households in low income countries. In contrast to vaccination of cattle, a high-value smallholder asset, there is a significant knowledge gap for the drivers of vaccine adoption of smallholder poultry. Newcastle disease virus (NDV) causes high mortality in chickens and is one of the greatest constraints to East African poultry production. To determine preferences and willingness to pay for NDV vaccines by chicken-owning households in Tanzania, we administered a survey with a contingent valuation activity to 535 households across six villages in Arusha, Singida, and Mbeya regions. Given the low current vaccination rate, we tested the null hypothesis that smallholder households do not value NDV vaccines and found overwhelming evidence that smallholders *do* value NDV vaccines. The willingness to pay (WTP) estimate was 5853 Tanzanian shillings ($2.64) to vaccinate ten chickens given the vaccine was protective for a period of three months. This estimate is about twice the market price reported by households in the study areas suggesting chicken-owning households value and benefit from NDV vaccines, but face other barriers to vaccination. Previous vaccination had the largest positive effect size on WTP suggesting smallholders observe benefits from vaccinating. In contrast to studies of vaccination of higher-cost cattle where off-farm income sources often drive willingness to pay, on-farm income was a driver of WTP for NDV vaccines suggesting different drivers affect protection of low-value livestock assets as compared to high-value assets.

## Highlights

• Willingness to pay signals low income households value Newcastle disease vaccines.
• Vaccination is valued despite poultry being a relatively low value asset.
• On-farm income is sufficient to drive willingness to pay (WTP).
• Prior vaccination increases WTP, implying vaccines are valued as being efficacious.

## 1 Introduction

Livestock play an important role in the food and economic security of smallholder households, defined as agricultural households with limited resource endowments relative to other farmers in the sector [1]. Livestock raised by smallholder farmers serve as an asset and source of income at the household level and, importantly, provide food for rural and urban consumers at the national level [2]. The Food and Agriculture Organization of the United Nations estimates that 1.5 billion people live in smallholder households worldwide, and in Africa and Asia, smallholders produce up to 80 percent of the total food supply [3]. Infectious disease is a significant constraint to livestock production in many developing regions [4], [5]; vaccination can be an effective risk management approach to minimize the burden of disease and increase livestock productivity. While governments, non-governmental organizations, and the private sector may play a role in vaccine development, production, and dissemination, the decision to vaccinate and the responsibility for the purchase and delivery of vaccines often falls to individuals, especially in the case of routine vaccination of endemic disease [6].

Previous studies of willingness to pay and adoption of livestock vaccines by smallholder farmers highlight the importance of household income as a driver of vaccination, especially in the case of high-value livestock such as cattle. Karanja-Lumumba et al. modeled factors affecting adoption of East Coast Fever vaccine by smallholder dairy farmers in Kenya, and found the vaccine was more likely to be adopted by relatively wealthy households with sources of off-farm income [7]. For agropastoralists in western Kenya, vaccine uptake for East Coast Fever was driven by the fraction of improved, higher-productivity exotic cattle breeds – reflecting a higher value resource – as well as off-farm income [8]. In a willingness to pay study of emergency and routine foot and mouth disease vaccination by pastoralists in Tanzania, Railey et al. found households with high levels of off-farm income and households with some income from selling crops in the previous season had higher willingness to pay for both emergency and routine vaccination [9]. In a study concurrent to the one described here, Campbell et al. identified determinants and barriers to adoption of Newcastle disease (ND) vaccines in Tanzania. Knowing someone who vaccinated and having a larger flock increased the odds of previous vaccination while using traditional medicines to treat or prevent ND decreased the odds of previous vaccination [10]. Notably, income in the previous month was not significantly associated with previous or recent vaccine use [10].

In contrast to vaccination of cattle, a high-value smallholder asset, there is a significant knowledge gap on the drivers of vaccine adoption of smallholder poultry. This is most relevant for highly contagious pathogens that cause high flock mortality, such as Newcastle disease virus (NDV). Despite NDV having been identified as the greatest constraint on poultry production in East Africa and the availability of effective vaccines, vaccination rates for smallholder poultry are low. In Tanzania, only 22% of households regularly vaccinate their chickens [11]. While chickens are kept by most rural households in the developing world [12] and contribute significantly to household food and economic security, they are also a much lower-value resource and the barrier to re-entry to ownership following loss of poultry is dramatically lower than for cattle.

As a consequence of the relatively low value of poultry, households may assume the risk of disease and replace chickens if necessary rather than pay for the vaccine. These decisions may be influenced by lack of information, perceptions and experience with vaccine efficacy, price sensitivity, or some degree of self-insurance such as keeping additional chickens to offset losses caused by disease. We test the null hypothesis that low-resource smallholder households in Tanzania do not value NDV vaccination by using a joint model of adoption and payment. We present the results of the study and discuss the findings in the context of drivers of household valuation of livestock.

## 2 Materials and methods

### 2.1 Contingent valuation

Multiple methods can be used to estimate the value of a good including revealed preference and stated preference techniques such as contingent valuation [13]. Revealed preference models involve observing marketplace behavior to learn about individual preferences. In contrast, stated preference methods create a hypothetical marketplace and are therefore ideal for commodities that are not exchanged in regular markets and for public goods such as control of disease or environmental improvement. Though NDV vaccines are exchanged in regular markets in Tanzania, the vaccination rate in Tanzania is low; consequently, NDV vaccines may represent a hypothetical good for many households. Contingent valuation (CV) was selected as an appropriate method for this research because it is a survey-based technique that estimates the value a person or household places on a single good (or several closely related goods) through their willingness to pay. A hypothetical marketplace also allows us to quantify differences in how households value traits of the vaccine and its delivery system. CV can be single or double-bounded, meaning the respondent is asked one or two questions respectively about their willingness to pay at a specific price. The double-bounded approach we employed was shown by Hanemann et al. (1991) to be asymptotically more efficient than the traditional single-bounded model where respondents are asked to give a yes or no response to a single bid [14].

### 2.2 Survey

**Fig. 1:**
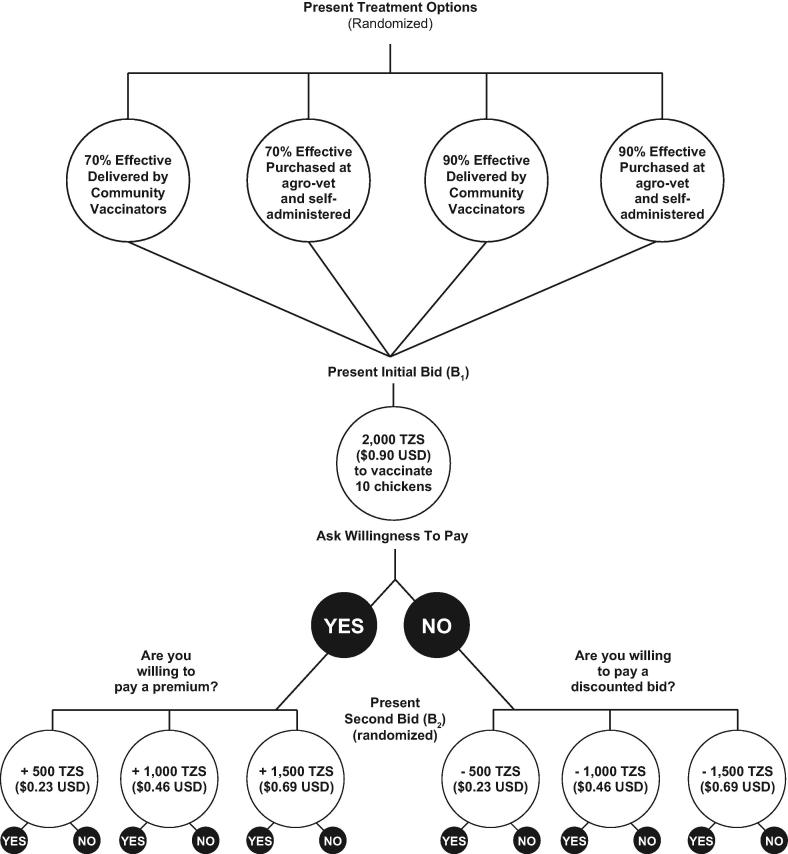
Description of bid amounts and treatments used in the WTP activity.

The survey was designed to measure potential predictors of adoption of NDV vaccines and willingness to pay for vaccines / vaccine services. The first section of the survey measured household demographics and socioeconomic status, as well as knowledge, attitudes, and practices with regards to chicken-keeping. The socioeconomic drivers of adoption are described by Campbell et al. [10]. The second portion of the survey consisted of a double-bounded contingent valuation activity where respondents within a household were asked if they were willing to pay 2000 Tanzanian shillings (TZS), equivalent to $0.90 USD, to vaccinate ten chickens for ND within the context of a specific, hypothetical scenario. Depending on the respondents’ answer to the first bid question, the amount of the second bid was increased (premium bid) or decreased (discounted bid) accordingly by a set amount randomized daily as shown in Fig. 1. The scenario described to respondents was randomized by two variables: vaccine efficacy (70% versus 90%) and delivery system (community vaccinators come to home and administer vaccine versus respondent purchases vaccine at an agro-veterinary shop). All survey questions were asked in terms of Tanzanian shillings, with USD equivalents presented using the exchange rate on June 30, 2017 of $1 USD = 2213 TZS [15].

The initial bid amount of 2000 TZS to vaccinate ten chickens was informed by estimating the distribution of willingness to pay using a pilot study. Twenty-five individuals were selected from each village using an outdoor transect walk or by intercepting every other person who passed a fixed point along a busy walkway. If the selected person consented to participate and had chickens, they were asked what amount they would be willing to pay to vaccinate ten chickens for ND with a vaccine that is protective for three months. These open-ended responses were collected as a reference baseline, but not used in the main analyses.

A quantity of ten chickens was selected for the activity because the cost of a single dose of vaccine might be less than 50 TZS, the lowest value coin in Tanzania, and because ten chickens is mathematically simple and only one less than the mean flock size of households nationally [16]. We intentionally did not specify within the scenario whether the vaccine was administered via drinking water (La Sota) or via eye drops (I-2). This decision had the benefit of making the scenario familiar and applicable to a respondent who had experience with either vaccine but limited the ability to learn about preferences by comparing the willingness to pay between the two vaccine types. To address manufacturer differences in vaccination schedule that require revaccination three to four times per year, we chose the most conservative time period for the contingent valuation activity and told respondents the vaccine would be protective for a period of three months. This corresponded with advice given by many Agricultural Officers we worked with that encourages farmers to vaccinate “every three months” which is often interpreted as a mandate to vaccinate sometime within the fourth month.

The survey was translated into Swahili and administered in-person to selected households by pairs of local research assistants, one male and one female. All surveys were administered between April and June 2017. Eligible households had a consenting adult at least 18 years old and currently owned local chickens (indigenous breed or crosses) or had owned them within the last six months. Respondents were informed that participation was voluntary and those choosing to participate provided oral consent. Household purchasing decisions such as buying vaccines may involve multiple household members, so household members were encouraged to discuss before making a final decision. The survey took about 45 min, and households were given one kilogram of sugar and a box of tea leaves upon completion of the survey to thank them for their time. This research was cleared by the Tanzanian Commission for Science and Technology (COSTECH) through permit No. 2018-32-NA-2015-213. The Washington State University Office of Research Assurances found the project exempt from the need for IRB review (#15068).

### 2.3 Study area

A multi-stage sampling approach was used to select households in six villages across three regions in Tanzania (Arusha, Singida, and Mbeya) with the goal of maximizing variation in poultry production practices, access to veterinary services, household demographics, and other variables with potential to influence willingness to pay for vaccines. At the village level, households were randomly selected using a census of heads of households provided by village governments as a sampling frame [17]. A total of 535 households were surveyed, which was reduced to 509 observations after data cleaning.

**Fig. 2:**
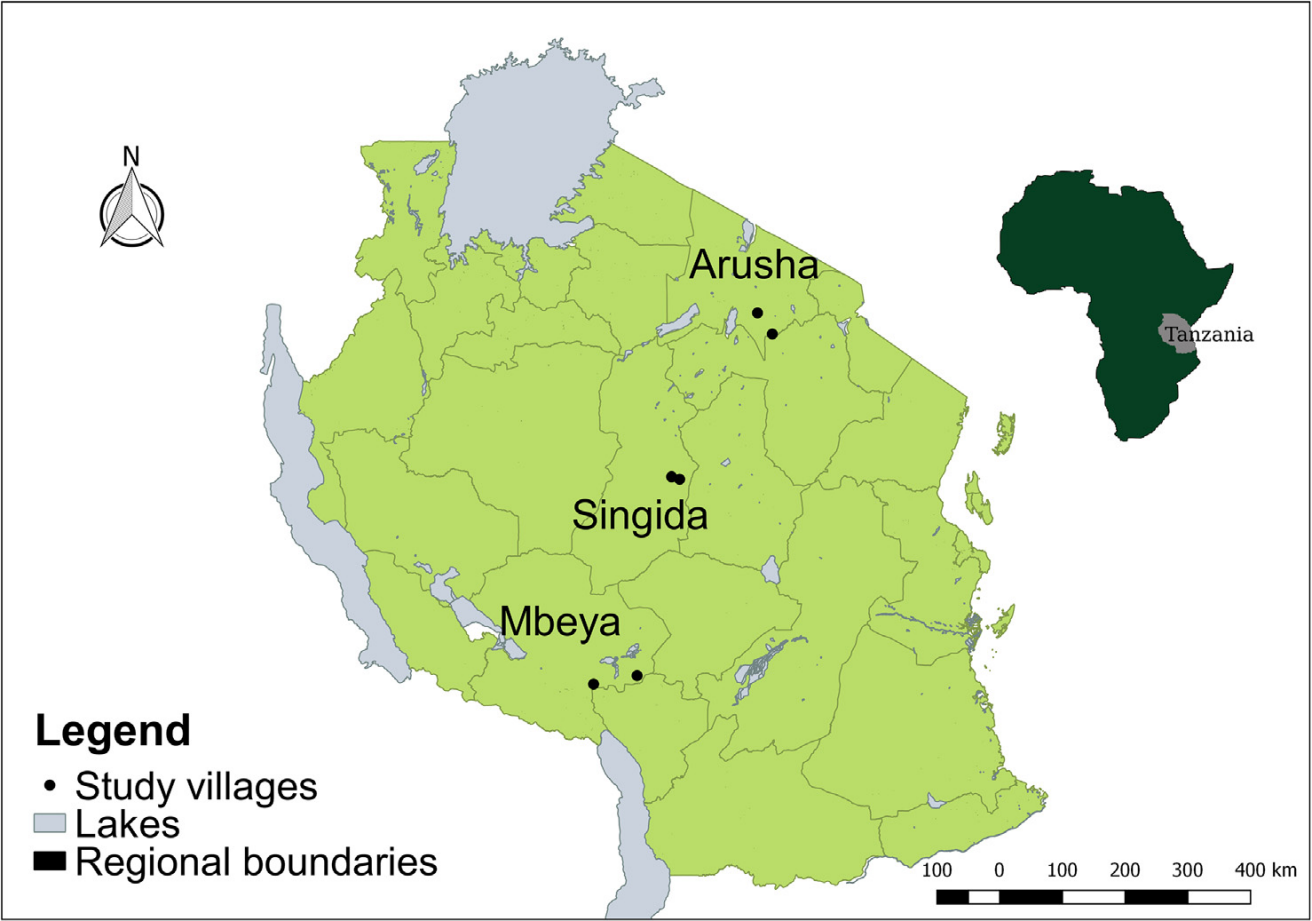
Map of study villages in Tanzania.

The three regions span Tanzania’s diversity in geography and climate and have different histories with regards to poultry production. The Arusha region in northern Tanzania is the home of ethnic groups such as the Maasai and Arusha that have traditionally focused on raising cattle and small ruminants rather than poultry. The Singida region in central Tanzania has a hot and dry climate and is known for successful poultry production and higher ND vaccination rates in part because of easy access to urban consumers by road and rail and fewer competing economic activities. Mbeya region has a cooler and rainier climate, and local governments have hosted some ND vaccination campaigns in the last ten years. In each region, one peri-urban village (<25 km from urban center) and one rural village (>25 km from urban center) were chosen for a total of six villages as shown in Fig. 2. The villages had between 1300 and 3000 residents and varied in their level of access to veterinary services [10].

### 2.4 Modeling willingness to pay

Contingent valuation was used to estimate willingness to pay parameters depending on two randomized treatments (efficacy and delivery system) and characteristics of the households such as income and previous vaccination. We estimated a double-bounded contingent valuation model with STATA, which is a commonly applied technique, following the maximum likelihood estimator described by Feldman-Lopez [18], [19]. Mathematical models for contingent valuation are provided in the Supplementary Materials (S1). After the parameters for the explanatory variables are estimated using maximum likelihood as described in S1, the estimated willingness to pay formula is linear, simply ${\tilde{z}}^{\prime }\hat{\beta }$, where z~' is the vector of values of the explanatory variables and $\hat{\beta }$ is the vector of parameters estimated by the maximum likelihood estimator.

The responses to the double-bounded CV questions give four possible discrete outcomes (D): (1) the household was not willing to purchase NDV vaccines even at the discounted price (“no”, “no” to both bids); (2) the household was not willing to purchase NDV vaccines at the initial price, but was willing to buy at the discounted price (“no”, “yes”); (3) the household was willing to purchase NDV vaccines at the initial price but not the increased, premium price (“yes”, “no”); or (4) the household was willing to purchase NDV vaccines at both the initial price and the premium price (“yes”, “yes”) [14]. Using the double-bounded model allows us to place the household’s WTP into one of four intervals: (−∞, B_D_), (B_D_, B_I_), (B_I_, B_P)_), or (B_P_, +∞) where B_D_, B_I_, and B_P_ are discounted, initial, and premium bids respectively. The bidding mechanism results in the following discrete outcomes:

(1) $$D=\left\{\begin{array}{ccc} 1 & WTP<B_{D} & \left(No,No\right)\\ 2 & B_{D}\leq WTP<B_{I} & \left(No,Yes\right)\\ 3 & B_{I}\leq WTP<B_{P} & \left(Yes,No\right)\\ 4 & B_{P}\leq WTP & \left(Yes,Yes\right) \end{array}\right)$$

where WTP is the household’s willingness to pay for NDV vaccine for ten chickens.

Model selection was performed with the aims of increasing log likelihood of the model, reducing multicollinearity of independent variables, and retaining significant terms (p < 0.1).

## 3 Results

### 3.1 Survey results

The consent rate in the survey was 99%, with 759 of the 766 households approached consenting to participate. Not all consenting households met the eligibility criteria, namely current or recent chicken ownership. Summary statistics for household variables are presented in Table 1. Across all regions, 91% of households believed they had seen NDV in their flock at some time. Seventy-seven percent of all households reported mortality of a third or more of their chickens within the last six months and 89% of these households believed NDV was the cause of mortality (356/400 households). Though these reports are not supported by diagnostic testing, high mortality is consistent with clinical signs of NDV and the high percentage of households reporting NDV indicates that it is perceived as a significant threat.

**Table 1:** Summary statistics for household variables with 95% confidence intervals (CI).

	Arusha	Mbeya	Singida	All regions
Self-reported NDV in flock (%)				
No	5 (2–9)	9 (6–14)	14 (9–20)	9 (7–12)
Yes	95 (91–98)	91 (86–94)	86 (80–91)	91 (88–93)

Aware of NDV vaccines (%)				
No	29 (23–36)	18 (13–24)	11 (7–17)	19 (16–23)
Yes	71 (64–77)	82 (76–87)	89 (83–93)	81 (77–84)

Previous ND vaccination (%)				
No	55 (47–62)	41 (34–48)	33 (26–40)	43 (39–47)
Yes	45 (38–53)	59 (52–66)	67 (60–74)	57 (53–61)
Knowledge score (M)^a^	38 (33–43)	46 (40–51)	55 (50–59)	46 (43–49)

Decision-maker gender (%)				
Male	16 (11–22)	35 (28–42)	52 (45–60)	34 (30–38)
Female	84 (78–89)	66 (58–72)	48 (40–55)	66 (62–70)

Decision-maker education				
No formal education	34 (27–42)	24 (18–31)	16 (11–22)	25 (21–28)
Primary school	60 (52–67)	67 (59–73)	72 (65–78)	66 (62–70)
Secondary and above	6 (3–11)	10 (6–15)	12 (8–18)	9 (7–12)
Tropical Livestock Units (M)	9 (7–11)	3 (1–4)	3 (2–4)	5 (4–6)
On farm income USD (M)	$104 (49–159)	$22 (9–36)	$14 (8–19)	$46 (27–52)

On farm income^b^				
None	41 (34–49)	71 (64–78)	75 (68–81)	63 (58–67)
Low-mid	29 (23–37)	20 (15–27)	19 (14–26)	23 (19–27)
High	29 (23–37)	8 (5–14)	6 (3–11)	14 (12–18)
Off farm income USD (M)	$44 (23–65)	$23 (15–32)	$25 (16–35)	$31 (23–39)

Off farm income^b^				
None	57 (49–64)	54 (47–61)	55 (48–63)	55 (51–60)
Low-mid	31 (25–39)	37 (30–44)	35 (28–43)	34 (30–39)
High	12 (8–18)	10 (6–15)	9 (6–15)	10 (8–13)
N	169	178	170	517

M refers to mean.

Figures may not add to 100 due to rounding.

a Percentage score on a five-question knowledge test.

b Household income in the last month: Low-Mid = 1–1,99,999 Tanzanian shillings (TZS) ($0.01–$90.36 USD); High = 2,00,000 TZS and up ($90.37 and up USD). Exchange rate: $1 USD = 2213 TZS.

Eighty-one percent of the households were aware of NDV vaccines and half the households reported previous use of NDV vaccines, but only 26% of households had vaccinated recently within the last four months as per manufacturer’s guidelines (95% CI: 22–30) by Campbell et al. [10]. Many households had an incomplete understanding of Newcastle disease and NDV vaccines as evidenced by an average knowledge score of 46% over five questions. See Supplementary Materials (S2) for knowledge questions. The main decision-maker for chickens in the household was a woman in 66% of households, though there was variation by region. Previous vaccination and knowledge scores were significantly higher in the Singida region than in the Arusha region, and the percentage of women decision-makers was significantly lower in Singida compared to Arusha. In Mbeya region, knowledge rates, percentage of previous vaccination, and percentage of female decision-makers fell between the figures for Arusha and Singida. Though chickens are sometimes referred to as “the woman’s cow” because women commonly make management decisions about chickens, the percentage of female decision-makers fell below 50% in Singida region. The majority of decision-makers for chickens within the households had completed primary school (66%), and a quarter of decision-makers had no formal education. Again, we saw differences between Singida and Arusha with decision-makers in Singida significantly more likely to have formal education than their counterparts in Arusha.

Total monthly income reported by the household in the last month is divided into on-farm income and off-farm income. On-farm income includes sale of crops, livestock, and animal products (See Supplementary Materials S3 for the break-down of sources of on-farm income). Off-farm income includes salaries, non-agricultural business earnings, remittances, and income from rent. There was no significant difference between mean on-farm income (M = $46 USD, SD = $220 USD) and mean off-farm income (M = $31, SD = $91 USD); t (516) = 1.47, p = 0.14) in the previous month across all regions. On-farm income was significantly higher in Arusha compared to the other two regions, with a mean of $104 USD compared to $22 in Mbeya and only $14 in Singida. Wealth in livestock was measured in Tropical Livestock Units (TLUs), a metric developed by the Food and Agriculture Organization of the United Nations, which allows for the combination of multiple species of livestock into a weighted measure representing total body weight and potential market value [20]. Arusha households had more wealth in livestock than households in the other two regions, owning a mean of nine TLUs compared to three in Mbeya and Singida.

### 3.2 Willingness to pay

The household responses to the bids in the double-bounded contingent valuation activity are summarized in Table 2. Eighty-seven percent of households responded “yes” to both the initial and the second, premium bid. The survey questions used in this activity are presented in Supplementary Materials S4.

**Table 2:** Contingent valuation double-bounded responses (proportions), n = 517.

	Second bid	
First bid	No	Yes
No	0.03	0.03
Yes	0.07	0.87

The parameter estimates of the explanatory variables in the preferred model are presented in Table 3. Maximum likelihood gives the parameter estimates, and the WTP formula is a linear function of the vector of explanatory variables multiplied by the vector of estimated parameters. Therefore, in a case with zero impact from explanatory variables, the WTP estimate is the constant, estimated at 4920. This can be interpreted as a household WTP of 4920 Tanzanian shillings, or $2.22 USD to vaccinate ten chickens for three months. Positively signed parameter estimates increase willingness to pay and negatively signed parameter estimates decrease willingness to pay. The magnitude of the change is expressed in Tanzanian shillings.

**Table 3:** Parameter estimates of the explanatory variables of mean household willingness to pay for ND vaccines.

Parameter	Parameter estimates
Previous ND vaccination	1646**
Knowledge score^a^	340*
Previous vaccination * knowledge	−34***
On farm income: none *(Reference)*^b^	
On farm income: low-mid^b^	966**
On farm income: high^b^	1581**
Singida * no education *(Reference)*	
Singida * primary education	−858**
Singida * secondary education and higher	−740
Small flock, ≤3 chickens^c^	773
Constant	4920***
Log likelihood	−250.71
N	509

Note: *10% significance level, **5% significance level, ***1% significance level.

No parameter estimate is calculated for reference levels.

a For one additional correct answer on a five-question test.

b Household income in the last month: Low-Mid = 1–1,99,999 Tanzanian shillings (TZS) ($0.01–$90.36 USD); High = 200,000 TZS and up ($90.37 and up USD). Exchange rate: $1 USD = 2213 TZS.

c Mixed results regarding statistical significance, in above model p = 0.12.

Previous NDV vaccination by the household increases willingness to pay for NDV vaccines and is the variable with the largest effect size. A higher knowledge score on the five true/ false questions about ND and vaccines is also associated with a higher willingness to pay. The effect size of the knowledge score is smaller than for previous vaccination but getting one additional correct answer is equivalent to a coefficient of 340, or a 340 TZS increase in willingness to pay. The effect of previous vaccination on willingness to pay varies depending on the knowledge score, and vice versa, as evidenced by a significant interaction term. The interaction term slightly decreases willingness to pay, but the overall effect of knowledge and previous vaccination experience on willingness to pay is positive, as seen by the sign of the coefficients on the main effects.

Having on-farm income in the last month increases willingness to pay. Households in a high bracket of on-farm income have a higher willingness to pay than households in the low-mid level brackets, and households with any level of on-farm income had higher willingness to pay than households without on-farm income in the previous month. Off-farm income was removed from the preferred model because it was not significant (p > 0.1).

Households in the Singida region with a decision-maker who completed primary education have a lower willingness to pay than households in Singida in which the decision-maker has no formal education or secondary/higher education. For context, an interaction effect of Singida households with highly educated decision-makers is included even though it is not significant to show a trend of lower willingness to pay with increasing education in Singida.

Households with extremely small flock sizes of three or fewer chickens (25th percentile for flock size) showed increased willingness to pay, but with mixed results regarding significance at the 10% level. The dummy variable for small flock size was left in the preferred model for reference, with a p-value of 0.12.

Variables considered but not included in the best model (p > 0.1) were delivery system; vaccine efficacy; vaccine cost per dose by village; decision-maker age, gender, and education level; whether the household knows someone who vaccinates; use of traditional medicine to treat ND; region; livestock owned in TLUs; whether the household believes the using NDV vaccine will lead to a larger flock; and off-farm income last month. All possible interactions between gender and region, education level and region, and gender and education level were considered as well as an interaction between delivery system and flock size. Correlations were tested for which informed the creation of interaction terms such as knowledge score and previous vaccination.

Using this model, willingness to pay for NDV vaccine for ten chickens given the vaccine is protective for a period of three months is estimated at 5853 TZS or $2.64 USD (Table 4).

**Table 4:** Contingent valuation estimation of WTP for NDV vaccine (USD/10 chickens/3 months), N = 509.

Mean (TZS)	Mean (USD)	95% CI (TZS)	95% CI (USD)
5853	$2.64	5022–6684	$2.27–$3.02

In addition to the willingness to pay estimate, we collected the price per dose paid by 227 households the last time they vaccinated. Sixty-six vaccinating households were removed because they received the vaccine for free through an organized program or from friends and family members. The mean market price paid was 274 TZS (95% CI 214–333), or $0.12 USD. This represents 119 households that used I-2 vaccine and 102 households that used La Sota vaccine. The mean cost per dose of 585 TZS estimated in the willingness to pay activity is about twice the mean market price of 274 TZS. As an internal check, respondents are asked after the willingness to pay questions whether they feel it is fair for a manufacturer to charge 60 TZS per chicken to vaccinate. Eighty percent of households (415/517) indicated they felt this was fair.

## 4 Discussion

The results of this study overwhelmingly suggest that smallholder households have a strong preference for NDV vaccines. This is supported by the high percentage of yes-yes responses to the willingness to pay bids, the mean willingness to pay estimate compared to market prices, and the positive role of previous vaccination experience in increasing willingness to pay. Eighty-seven percent of households said yes to both the initial bid of 2000 TZS to vaccinate ten chickens and the second, higher premium bid. The mean willingness to pay bid is about twice the mean market price actually reported by households within the study, which further supports strong desire for NDV vaccination. Consequently, we reject the stated hypothesis that smallholder farmers do not value NDV vaccines. This is consistent with a contingent valuation study for Gumboro and Newcastle disease vaccine programs in Ethiopia which showed that farmers recognized the value of vaccine programs and were willing to pay for them [21].

Contingent valuation is a way to understand individual preferences and estimate value for a good using a monetary scale and therefore may not translate perfectly into comparisons with market behavior or market prices but there are ways to address validity concerns and avoid unreliable results. These include achieving high survey response rates, describing the good accurately, providing a realistic and believable scenario, and valuating goods with which respondents have some level of familiarity [13], [22]. When validity concerns are properly addressed, contingent valuation is a useful method for estimating the value consumers or producers place on a good, and it is now being applied globally [22], [23]. This study successfully avoided some common validity concerns inherent to contingent valuation by achieving an extremely high survey consent rate of 99%, describing the vaccine accurately, and providing two realistic delivery scenarios. One area of concern is the 19% of survey respondents who indicated they were not aware of NDV vaccines because valuating unfamiliar goods is associated with error. If this error is biased or nonrandom, it can affect the willingness to pay estimate [13]. Given the 90% and above coverage in Tanzania for many human immunizations [24], respondents who are not aware of NDV vaccines likely have a basic understanding of human vaccines from personal experience, which makes the concept of a livestock vaccine more familiar.

Previous NDV vaccination was the most influential driver of willingness to pay in our study, increasing a household’s willingness to pay by about a quarter of the mean willingness to pay. This can be interpreted as a household’s satisfaction or experience with the product; households that tried NDV vaccines valued them more than households without similar experience. This is consistent with literature indicating that NDV vaccines are safe and effective for use in smallholder chicken production [11], [25], [26]. The strong preference for NDV vaccines contrasts with studies by Railey et al. and Kairu-Wanyoike in which smallholder willingness to pay for routine foot and mouth disease vaccines and contagious bovine pleuropneumonia vaccines for cattle in Tanzania and Kenya, respectively, was tempered by concerns about vaccine efficacy and safety [9], [27]. The high impact of previous NDV vaccination on mean willingness to pay suggests that smallholders are able to experience first-hand a notable reduction in death loss, consistent with NDV representing a major cause of chicken mortality in East Africa.

Households with any level of on-farm income in the previous month had increased willingness to pay for NDV vaccines while off-farm income was not significant in the preferred model. The importance of on-farm income such as crop or livestock sales but not off-farm income such as business earnings as a driver of willingness to pay for chicken vaccines contrasts with willingness to pay for cattle vaccines, which is often positively influenced by increased wealth or sources of off-farm income [7], [8], [9]. The significance of on-farm income suggests households do not necessarily need to have a source of income outside of their agricultural activities to afford less costly NDV vaccines. Since 45% of households reported some off-farm income in the previous month, it may also suggest that households do not perceive price to be a barrier to vaccination and prefer to utilize on-farm income sources when they invest in protecting their chickens through vaccination. Many respondents verbally justified their willingness to pay decision by stating they would be prepared to sell one of their chickens to protect the hypothetical nine remaining. The average price received for selling one chicken by households in this study was 6900 TZS, or $3.12 USD. Households need some cash flow, as evidenced by the increase in willingness to pay for households with *any* level of on-farm income in the previous month as compared to households with no on-farm income in the previous month. Regardless of whether households are more dependent on livestock or crops, on-farm income is likely to be seasonal, raising the concern that during certain times of year, households may not have the cash on hand to purchase NDV vaccines. It highlights the vulnerability of households without income in the previous month due to seasonality, poverty, or other circumstances. Sale of a yearly subscription of vaccine by a trusted administrator to be paid for during periods of high income could be one way to address the challenge of seasonal income.

The level of education of the primary decision-maker for chickens was not significantly correlated with willingness to pay, but the interaction term of Singida region and primary education was significant. This term decreased willingness to pay compared to the reference level of no formal education interacted with Singida. This decreased willingness to pay may be caused by the increased familiarity with ND vaccines of households in Singida and the fact that decision-makers with some formal education may be more likely to know and remember actual market prices for ND vaccines, which are lower than the mean willingness to pay estimates. In a similarly structured study, Railey et al. found that formal education had no effect on willingness to pay of Tanzanian households for emergency foot and mouth disease vaccines for cattle [9]. In the case of routine foot and mouth vaccines, willingness to pay was higher for households where the head of household had no formal education compared to some formal education. These studies support the idea that a more educated and experienced buyer may be more scrutinizing and less likely to over-pay when purchasing vaccines.

Households were not sensitive to vaccine efficacy at the treatment levels described in the contingent valuation activity (70% versus 90% effective) or to the delivery system (community vaccinators versus self-purchase and administration). Some respondents may have found the concept of vaccine efficacy confusing. To address this, enumerators explained 70% efficacy as the vaccine protecting seven out of ten chickens in the event of a ND outbreak. Considering the inherent risk in keeping chickens with threats including disease, theft, and predation, the difference between 70% protection and 90% protection from disease may be perceived as minimal compared to the actual risks farmers face. Community vaccination, a delivery system in which livestock vaccines are administered on a pay-per-service basis to households by trained community vaccinators, has been proposed as a sustainable model for ND control in rural areas. The lack of difference in willingness to pay between delivery systems may suggest that either system would be viable for most households, but may also be a result of the way the willingness to pay activity was structured. Self-purchasing involves higher transaction costs than community vaccination, such as transportation to the place where the vaccines are sold and being forced to buy vaccines in larger quantities than most smallholders need (100 doses is a common minimum package size). Clearly mentioning and quantifying the transaction costs within the willingness to pay activity may have given clearer results about preferences between delivery systems. Additionally, and importantly, none of the study villages had a current and functional community vaccination system beyond informal co-operation by members of chicken-raising groups, which raises validity concerns if respondents did not perceive community vaccination to be a realistic delivery system.

One shortcoming of this study is the inability to address preferences between the two vaccine types, La Sota and I-2. By combining the two similar vaccines into a single hypothetical good, we assured the product was familiar to those with experience with either vaccine type, but we cannot comment on whether smallholder farmers value unique characteristics such as thermotolerance or administration method enough for their willingness to pay to be affected. Learning more about the underlying factors driving the choice between the two vaccine types is an area worthy of future investigation.

Controlling livestock disease through vaccination increases the health and productivity of livestock and allows smallholder households in developing countries to maximize the economic and nutritional benefits they receive from the livestock they own. This study demonstrates that low-resource households have a strong preference for vaccines that allow them to protect a low-value asset. A strong preference for vaccines in conjunction with relatively low rates of vaccination and the absence of a significant correlation between last month’s income and previous use of vaccines suggests households are facing barriers external to the household. With the knowledge that smallholder households overwhelming value and perceive benefits to using NDV vaccines, attempts to increase vaccine coverage should focus on other limitations smallholders face when purchasing and administering vaccines such as poor availability of reliable vaccines in rural areas due to supply chain inefficiencies opportunity costs, and high transaction costs especially for households with small flock sizes.

## Conflict of interest statement

The authors declare that they have no competing interests.
