# Supplementary material for: Drivers of vaccination preferences to protect a low-value livestock resource: Willingness to pay for Newcastle disease vaccines by smallholder households
Source: Vaccine. 2019 Jan 3;37(1):11–8. doi: 10.1016/j.vaccine.2018.11.058 (PMC6290109; doi:10.1016/j.vaccine.2018.11.058)
Supplement: Supplementary data 1 [file mmc1.docx]

**Supplementary Materials S1**

**Mathematical models for willingness to pay contingent valuation activity**

A simple, theoretical model shows willingness to pay as a function of explanatory variables and an error term for household $h$:

${WTP}_{h}\left( z_{h},u_{h} \right)=z_{h}\beta+u_{h}$ ( 1 )

where $z_{h}$ is a vector of explanatory variables, $\beta$ is a vector of parameters, and $u_{h}$ is an error term. By estimating β, we can estimate WTP depending on the values that we give to vector z. Given the goal of understanding determinants of household WTP for ND vaccine, identifying explanatory variables that make up vector z is a valuable output of modeling CV.

The true value of β is not known but can be estimated as $\hat{\beta}=-\frac{\hat{\alpha}}{\hat{\delta}}$ where$\hat{\alpha}$ is the vector of coefficients associated to each one of the explanatory variables and $\hat{\delta}$ is the coefficient for the variable capturing the amount of the bid [1].

The responses to the double-bounded CV questions give four possible outcomes: 1) the household was not willing to purchase ND vaccines even at the discounted price (“no”, “no” to both bids); 2) the household was not willing to purchase ND vaccines at the initial price, but was willing to buy at the discounted price (“no”, “yes”); 3) the household was willing to purchase ND vaccines at the initial price but not the increased, premium price (“yes”, “no”); 4) the household was willing to purchase ND vaccines at both the initial price and the premium price (“yes”, “yes”) [2]. Using the double-bounded model allows us to place the household’s WTP into one of four intervals: (- ∞, B_D_), (B_D_, B_I_), (B_I_, B_P)_), or (B_P_, + ∞) where B_D_, B_I_, and B_P_ are discounted, initial, and premium bids respectively. The bidding mechanism results in the following discrete outcomes:

$D=\left\{ \begin{aligned} \begin{matrix} 1 WTP< B_{D} (No, No) \\ 2 B_{D}\leq WTP< B_{I} (No, Yes) \\ 3 B_{I}\leq WTP< B_{P} (Yes, No) \end{matrix} \\ 4 B_{P}\leq WTP (Yes, Yes) \end{aligned} \right.$ ( 2 )

where WTP is the household’s willingness to pay for ND vaccine for ten chickens.

$$\text{WTP }\text{< }B_{D} (No, No)$$

As outlined by Lopez-Feldman, we define $y_{h}^{1}$ and $y_{h}^{2}$ as the dichotomous variables that capture the response to the first and second closed questions, ${z'}_{h}$ as a vector with the values for the explanatory variables of the household, $B_{1}$ and $B_{2}$ as the initial and second (premium or discount) bids, respectively. The function that needs to be maximized in order to find the parameters of the double-bounded CV model is:

$\sum_{h=1}^{N} \left[ \begin{aligned} d_{h}^{sn} ln\left( Ф\left( {z^{'}}_{h}\frac{\beta}{\sigma}-\frac{B^{1}}{\sigma} \right)-Ф\left( {z^{'}}_{h}\frac{\beta}{\sigma}-\frac{B^{2}}{\sigma} \right) \right)+d_{h}^{ss} ln\left( Ф\left( {z^{'}}_{h}\frac{\beta}{\sigma}-\frac{B^{2}}{\sigma} \right) \right) \\ +d_{h}^{ns} ln\left( Ф\left( {z'}_{h}\frac{\beta}{\sigma}-\frac{B^{2}}{\sigma} \right)-Ф\left( {z'}_{h}\frac{\beta}{\sigma}-\frac{B^{1}}{\sigma} \right) \right)+d_{h}^{nn} ln\left( 1-Ф\left( {z'}_{h}\frac{\beta}{\sigma}-\frac{B^{2}}{\sigma} \right) \right) \end{aligned} \right]$ ( 3 )

where $d_{i}^{sn}$,$d_{i}^{ss}$,$d_{i}^{ns}$,$d_{i}^{nn}$ are indicator variables that take on the value of one or zero depending on the relevant case for each household. By maximizing the function, we can directly obtain $\hat{\beta}$ and $\hat{\sigma}$ in order to estimate WTP [1]. The formula to predict WTP is simply $\tilde{z}'\hat{\beta}$, where $\hat{z}$ is the vector of values of the explanatory variables and $\hat{\beta}$ is the vector of parameters estimated by the maximum likelihood estimator.

**Citations**

[1] Lopez-Feldman A. Introduction to Contingent Valuation Using Stata. Apl. en Econ. y Ciencias Soc. con Stata, College Station, TX: Stata Press; 2012. doi:10.1258/095646202760029804.

[2] Hanemann M, Loomis J, Kanninen B. Statistical efficiency of double-bounded dichotomous choice contingent valuation. Am J Agric Econ 1991:1255–63. doi:10.2307/1242453.
