# Supplementary material for: Drivers of vaccination preferences to protect a low-value livestock resource: Willingness to pay for Newcastle disease vaccines by smallholder households
Source: Vaccine. 2019 Jan 3;37(1):11–8. doi: 10.1016/j.vaccine.2018.11.058 (PMC6290109; doi:10.1016/j.vaccine.2018.11.058)
Supplement: Supplementary data 2 [file mmc2.docx]

| **SECTION K**: **KNOWLEDGE** |
| --- |
| *Answer the following questions.* |
| **K1** A vaccine can help a sick chicken to get better. |
| 1 = Yes  2 = No  999 = Don’t know |
| **K2** The Newcastle vaccine must be given once per year. |
| 1 = Yes  2 = No  999 = Don’t know |
| **K3** Newcastle disease has no treatment, it only has a vaccine. |
| 1 = Yes  2 = No  999 = Don’t know |
| **K4** The Newcastle vaccine protects chickens against all illnesses. |
| 1 = Yes  2 = No  999 = Don’t know |
| **K5** The Newcastle vaccine may not be effective if it isn’t stored properly. |
| 1 = Yes  2 = No  999 = Don’t know |

**Supplementary Materials S2**

**Five question knowledge score**

Score was calculated by giving one point for each correct response, and no points for an incorrect or “don’t know” response. The correct responses were divided by 5 to give a proportional knowledge score.
