# Supplementary material for: Drivers of vaccination preferences to protect a low-value livestock resource: Willingness to pay for Newcastle disease vaccines by smallholder households
Source: Vaccine. 2019 Jan 3;37(1):11–8. doi: 10.1016/j.vaccine.2018.11.058 (PMC6290109; doi:10.1016/j.vaccine.2018.11.058)
Supplement: Supplementary data 3 [file mmc3.docx]

**Supplementary Materials S3**

**Sources of on-farm income**

On farm income was calculated by summing the following sources of income. Income from selling chickens and eggs makes up only 9% of the total mean household income reported last month on average, suggesting on-farm income is not endogenous to the question of willingness to pay for ND vaccines.

|  | **Mean** | **Standard error** | **% of mean total household income** |
| --- | --- | --- | --- |
| Crop sales | 65,000 | 12,000 | 64% |
| Non-chicken livestock sales | 27,000 | 14,000 | 26% |
| Non-chicken animal product sales | 1,000 | 300 | 1% |
| Chicken sales | 7,000 | 1,000 | 7% |
| Egg sales | 2,000 | 600 | 2% |
| TOTAL | 102,000 | 20,000 |  |

**Fig 1 Break-down of sources of on-farm in come in the previous one month in Tanzanian shillings (TZS)**
