# Supplementary material for: Drivers of vaccination preferences to protect a low-value livestock resource: Willingness to pay for Newcastle disease vaccines by smallholder households
Source: Vaccine. 2019 Jan 3;37(1):11–8. doi: 10.1016/j.vaccine.2018.11.058 (PMC6290109; doi:10.1016/j.vaccine.2018.11.058)
Supplement: Supplementary data 4 [file mmc4.docx]

**Supplementary Materials S4**

**Willingness to pay survey questions**

Delivery system (1 OR 2)

Percent efficacy (70% OR 90%)

Change in bid (-1,500, -750, -500, +500, +750, +1500). See chart below. The initial bid is always 2,000/=.


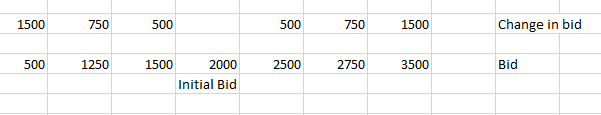


ENGLISH

***(Read introduction)*** This is the final section in the questionnaire. We will ask you what you are willing to pay for Newcastle disease vaccine. We are not selling or providing vaccine, we are conducting research to understand the need for vaccines in Tanzania. If you have any questions, please ask. It is fine to discuss with family members before answering.

**Delivery system 2:** The vaccine is available at the agrovet shop, and you need to go and purchase it yourself. The vaccine will protect your chickens against Newcastle disease for three months. The vaccine will be mixed into the chickens’ drinking water or delivered via eyedrops. Please remember the cost of the vaccine is in addition to other living costs that your household spends. **Suppose the vaccine has a ___% efficacy and protects each vaccinated chicken for three months.** For this example, assume you have ten chickens to vaccinate.

**Delivery System 1:** The vaccine service will be offered by trained community vaccinators who will come to your home. The vaccine will protect your chickens against Newcastle disease for three months. The vaccine will be mixed into the chickens’ drinking water or delivered via eyedrops. Please remember the cost of the vaccine is in addition to other living costs that your household spends. **Suppose the vaccine has a ___% efficacy and protects each vaccinated chicken for three months.** For this example, assume you have ten chickens to vaccinate.

| Did you understand the details of this vaccination program? Yes / No |
| --- |
| *If respondent answers NO to question 1, go back and explain - until you receive the answer YES.* |
| If the cost to vaccinate TEN chickens is ___tsh, are you willing to pay? |

SWAHILI

***(Asome mtafiti)*** Hiki ni kifungu cha mwisho katika maswali. Tutakuuliza kuhusu utayari wako wa kulipa kwa ajili ya chanjo za kideri. Hatutengenezi wala hatuuzi chanjo, tufanya utafiti huu ili tuelewe mahitaji ya chanjo hapa Tanzania. Kama una swali lolote, tafadhali uliza. Inaruhusiwa kufanya majadiliano na familia ili kupata uelewe.

**Njia ya pili ya utoaji:** Chanjo inapatikana kwenye duka la dawa za mifugo, na ina bidhii uende kununua mwenyewe. Kuchanja mara tatu kwa mwaka ni muhimu kwa kuzuia ugonjwa. Chanjo hii itawapa kinga kuku wako dhidi ya maradhi ya mdondo/kideri. Chanjo itakoyotolewa ni ya kunywesha kwenye maji au matone kwenye jicho.

Huduma hii itakuwa ya malipo kwa kaya ambapo itatakiwa kulipa ili upate faida. Tafadhali kumbuka hii ni nyongeza ya gharama zingine sa maisha ambayo kaya inagharimia. **Tuseme chanjo ina uwezo wa kukinga kuku kwa asilimia____na inafanya kazi kwa kipindi cha miezi mitatu.** Kwa maneno mengine, ugonjwa ukija, asilimia __ ya kuku watakingwa na hawataumwa mdondo / kideri. Kwa ajili ya mafano huu, tuseme una kuku kumi nyumbani.

**Njia ya kwanza ya utoaji:** Huduma ya chanjo itatolewa na wanajamii waliofundishwa. Watakuja nyumbani kwako mara tatu kwa mwaka na watachanja kuku wote. Kuchanja mara tatu kwa mwaka ni muhimu kwa kuzuia ugonjwa. Chanjo hii itawapa kinga kuku wako dhidi ya maradhi ya mdondo/kideri. Chanjo itakoyotolewa ni ya kunywesha kwenye maji au matone kwenye jicho.

Huduma hii itakuwa ya malipo kwa kaya ambapo itatakiwa kulipa ili upate faida. Tafadhali kumbuka hii ni nyongeza ya gharama zingine sa maisha ambayo kaya inagharimia. **Tuseme chanjo ina uwezo wa kukinga kuku kwa asilimia____na inafanya kazi kwa kipindi cha miezi mitatu.** Kwa maneno mengine, ugonjwa ukija, asilimia __ ya kuku watakingwa na hawataumwa mdondo / kideri. Kwa ajili ya mafano huu, tuseme una kuku kumi nyumbani.

| Je, umeelewa maelezo ya huduma ya chanjo? |
| --- |
| *Kama jibu ni hapana, rudi na eleza mpaka jibu liwe ndiyo.* |
| Je, kama gharama ya chanjo ni **sh____**kwa kuku **KUMI**, uko tayari kulipa? |
